# Somatic regional oxygen saturation as an early marker of intra-abdominal hypertension in critically ill children: a pilot study

**DOI:** 10.3906/sag-1903-201

**Published:** 2020-04-09

**Authors:** Özden Özgür HOROZ, Nagehan ASLAN, Dinçer YILDIZDAŞ, Yasemin ÇOBAN, Yaşar SERTDEMİR, Awni AL-SUBU

**Affiliations:** 1 Department of Pediatrics, Division of Pediatric Critical Care Medicine, Çukurova University Faculty of Medicine, Adana Turkey; 2 Department of Biostatistics, Çukurova University Faculty of Medicine, Adana Turkey; 3 Department of Pediatrics, Division of Pediatric Critical Care Medicine,University of Wisconsin School of Medicine and Public Health, Madison, WI USA

**Keywords:** Intraabdominal hypertension, NIRS, mesenteric rSO2, central venous oxygen saturation, vasoactive inotropic score, pediatric intensive care unit

## Abstract

**Background/aim:**

Intraabdominal hypertension is a common clinical condition with high mortality and morbidity in pediatric intensive care units. The aim of this study was to test the feasibility of regional tissue oxygenation (rSO2) measurement using near-infrared spectroscopy and to assess the correlation between rSO2 and perfusion markers of intraabdominal hypertension in high-risk pediatric patients.

**Materials and methods:**

In this prospective observational cohort study in a tertiary pediatric intensive care unit in Çukurova University Faculty of Medicine, a total of 31 patients who were admitted between May 2017 and May 2018 with a risk of intraabdominal hypertension were included. Mesenteric and renal rSO2 measurements were taken and correlations with other tissue perfusion markers including mean arterial pressure, pH, lactate, intraabdominal pressure, abdominal perfusion pressure, mixed venous oxygen saturation, vasoactive inotropic score were assessed. Intraabdominal pressure was measured as ≥10 mmHg in 15 patients (48.3%) and these patients were defined as the group with intraabdominal hypertension.

**Results:**

In the group with intraabdominal hypertension, mixed venous oxygen saturation was lower (P = 0.024), vasoactive inotropic score was higher (P = 0.024) and the mean abdominal perfusion pressure value was lower (P = 0.014). In the ROC analysis, the mesenteric rSO2 measurement was the best parameter to predict intraabdominal hypertension with area under the curve of 0.812 (P = 0.003) 95% CI [0.652–0.973].

**Conclusion:**

Monitoring of mesenteric rSO2 is feasible in patients at risk for intraabdominal hypertension. Moreover, both mesenteric regional oxygen and perfusion markers may be used to identify pediatric patients at risk for intraabdominal hypertension.

## 1. Introduction

Intraabdominal hypertension (IAH) and abdominal compartment syndrome (ACS) are associated with high mortality and morbidity rates in children. The World Society of the Abdominal Compartment defines pediatric IAH as having intraabdominal pressure (IAP) > 10 mmHg in continuous or repeated measures with new or worsening organ dysfunction that can be attributed to elevated IAP [1]. Elevated IAP affects both abdominal and extraabdominal organs. It can cause a variety of effects on the functions of the gastrointestinal, renal, respiratory, cardiovascular, and central nervous systems [2]. The vicious cycle of poor perfusion starts and increasing IAP leads to ischemic cellular necrosis [3]. The incidence of IAH is 12.6% and the prevalence is 43.9% in critically ill children in pediatric intensive care units [4,5]. The mortality of ACS in critically ill children ranges from 16% to 100% in various studies [4,6,7]. Abdominal perfusion pressure (APP) is the difference between the mean arterial pressure (MAP) and the IAP and can be thought of as an abdominal analog of cerebral perfusion pressure. Optimum APP level in children is not yet defined. Children have lower MAP levels than adults and therefore have an increased risk of developing multiple organ failure in lower IAP levels than adults [8,9]. Risk factors for IAH were defined by the consensus published by WSACS [2]. 

Near-infrared spectroscopy (NIRS) is a technique that evaluates regional tissue oxygenation (rSO2) by noninvasive methods [10]. It measures cerebral or somatic rSO2 and can be used as a marker of hypoperfusion. There is no absolute value reported in the literature yet, though cerebral rSO2 < 50% indicates severe brain injury. Both cerebral and somatic measurements <30%, or a 20% decrease from baseline, are considered urgent and it points to ischemia [11]. 

In this study, we aimed to determine the relationship of rSO2 measurements with NIRS to other perfusion markers in terms of detecting intraabdominal hypertension in patients in the risk groups.

## 2. Materials and methods

Patients who were admitted to the Çukurova University Faculty of Medicine, Balcalı Hospital Pediatric Intensive Care Unit between May 2017 and May 2018 were evaluated prospectively. Patients with risk factors for IAH, defined using the WSACS consensus decision, were included the study (1). Table 1 shows the risk factors for IAH. Mesenteric and renal rSO2 measurements were performed. Other tissue perfusion markers such as MAP, APP, lactate, IAP, capillary refilling time, central mixed venous oxygen saturation (ScvO2), acidosis, hypothermia, sepsis, coagulopathy, mechanical ventilation status, pediatric logistic organ dysfunction (PELOD) score and pediatric risk of mortality score (PIM) II score, pediatric risk of mortality (PRISM) III score were recorded. Ejection fraction (EF) was measured and vasoactive inotropic score (VIS) was calculated. The VIS was calculated as follows: [dopamine (µg/kg/min)] + [dobutamine (µg/kg/min)] + [10,000 × vasopressin (U/kg/min)] + [10 × milrinone (µg/kg/min)] + [100 × epinephrine (µg/kg/min)] + [100 × norepinephrine (µg/kg/min)] [12]. 

**Table 1:** Risk factors for IAH in children defined by WSACS.

Risk factors
Abdominal surgery
Congenital abdominal wall defects
Major traumas, burns
Increased intraluminal contents
Gastroparesis
Ileus
Volvulus
Hirschprung’s disease
Increased abdominal contents
Acute pancreatitis
Intraabdominal infections/abscess
Intraabdominal tumors
Peritoneal dialysis
Laparoscopy
Extracorporeal membrane oxygenation
Ascites
Intestinal/kidney transplantation
Wilm’s tumor/Burkitt’s lypmhoma
Capillary leak/fluid resuscitation
Sepsis/septic shock
Acidosis
Hypothermia
Massive fluid resuscitation
Positive fluid balance
Polytransfusion
Other risk factors
Obesity or increased body mass index
PEEP > 10
Coagulopathy
PRISM III score > 17
>30 plateau pressure for ventilated patients
Pneumonia
Mechanical ventilation

**Figure 1:**
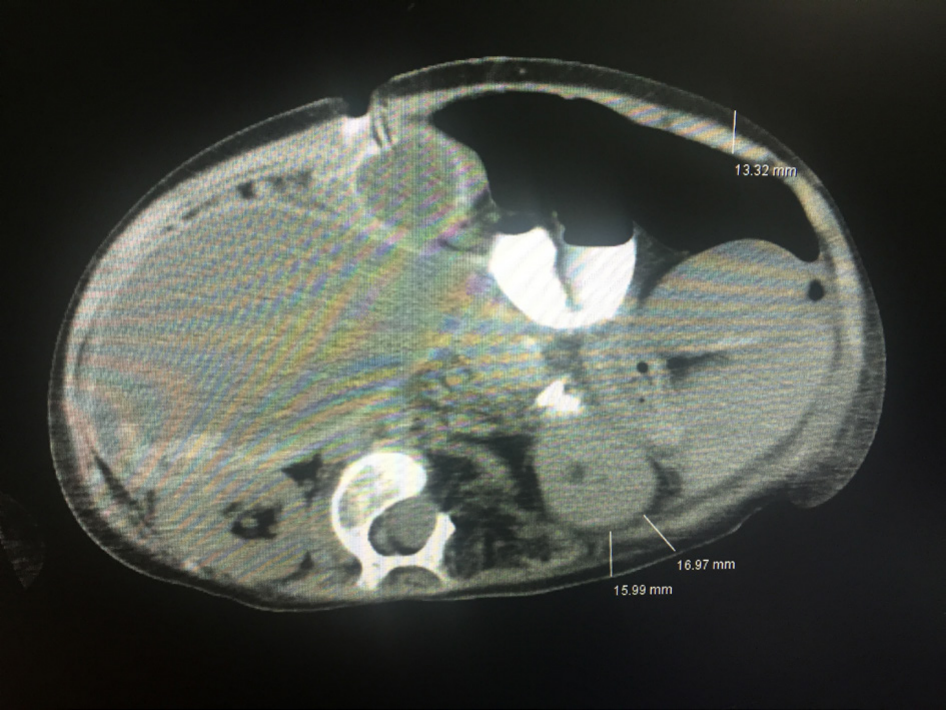
Showing the locations of measurement of posterior, anterior, and posterolateral abdominal wall thickness, 120 month-old, 23 kg patient.

Mesenteric and renal rSO2 measurements were performed using the NIRS (Cerebral OxyAlertTM, Somanetics Invos 5100C, Somanetics Corporation, Troy, MI, USA) Pediatric NIRS sensors were placed on two locations: just below the umbilicus on the anterior abdominal wall for mesenteric rSO2 and the T10-L1 dorsal lateral region for renal rSO2. In children aged 6 years and over 25 kg, tomographic abdominal wall thickness was measured for the sensitivity of NIRS sensors (Figure 1). To avoid the effect of sampling depth on the NIRS measurement, we excluded patients over 25 kg as abdominal wall thicknesses correlated better with the weight of the subjects than age [13].

Capillary refilling time was measured by the same pediatrician on sternum or forehead by applying pressure with soft pad of index finger for 5 s to blanch the area and then releasing finger to note the return of circulation with the help of wall clock.

Mesenteric rSO2, renal rSO2 and MAP measurements were performed continuously but recorded 4 times at 6-h intervals and the mean value was calculated. The indirect measurement method was used for IAP measurement as recommended by WSACS. Intraabdominal pressure was measured 4 times at 6-h intervals. One milliliter per kilogram (minimum 3, maximum 25 mL) saline was administered to the bladder with a foley catheter. After waiting for 30–60 s, IAP measurement was made at end-expiration in the supine position. Abdominal perfusion pressure was calculated using the formula (MAP-IAP).

Çukurova University Faculty of Medicine clinical research ethics committee approved the study. Written informed consent was obtained from the families of the patients.

## 3. Statistical analysis

All analyses were performed using IBM SPSS Statistics Version 19.0 statistical software package. Categorical variables were expressed as numbers and percentages, whereas continuous variables were summarized as mean and standard deviation and as median and minimum–maximum where appropriate. For comparison of continuous variables between two groups, Mann–Whitney U test was used. A receiver operator characteristic (ROC) curve analysis was performed in order to identify the optimal cut-off point of IAH. The statistical level of significance for all tests was considered to be 0.05.

## 4. Results and discussion

**Figure 2:**
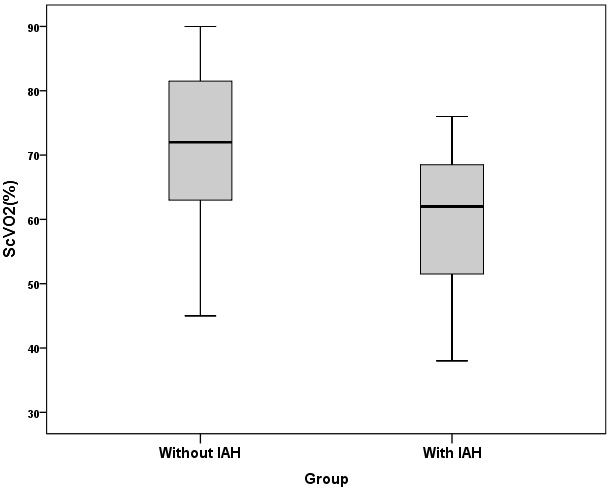
ScvO2 values of the two groups.

**Figure 3:**
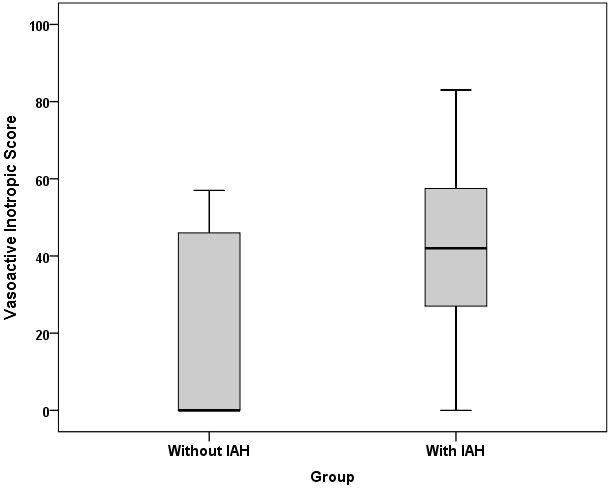
Vasoactive inotropic score values of the two groups.

**Figure 4:**
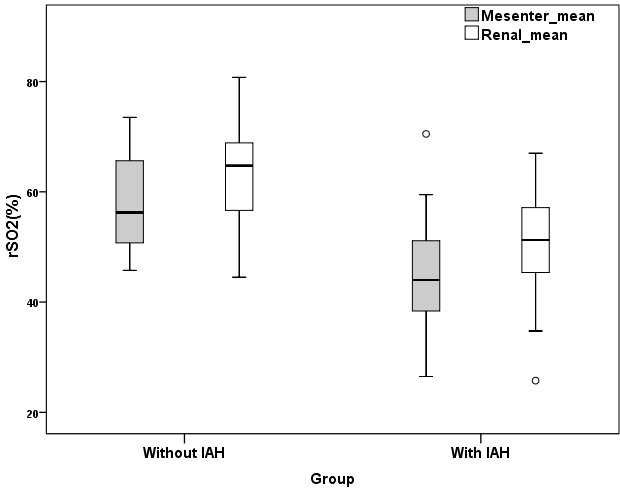
Mean rSO2 mesenteric and rSO2 renal values of the two groups.

Thirty-one patients were included in this study, 11 patients (35.4%) were female. The mean age was 60.8 ± 47.9 months (Range: 2–126 months). The admission diagnoses of the patients in study are shown in Table 2. Intraabdominal pressure was measured ≥10 mmHg in 15 patients (48.3%) and these patients were defined as the group with IAH. There were no significant differences in terms of age, sex, body weight, PIM 2, PRISM III, urinary output, BUN, creatinine level, hypothermia, coagulopathy, MAP between with IAH and without IAH groups. There was a significant difference between the two groups in terms of capillary refilling time, mean APP, mean lactate and mean rSO2 mesenteric, mean rSO2 renal values, ScvO2, VIS, and EF (Table 3). Acidosis was detected in 73.3% (P = 0.183), and sepsis was detected in 73.3% of the group with IAH (P = 0.063). Fourteen patients in the with IAH group (93.3%) were mechanically ventilated (P = 0.018) and 92.8% of them were receiving sedation and analgesia. In patients with IAH, PELOD score was higher than that in the without IAH group (P = 0.086). The mean capillary refilling time was 3.8 ± 1.1 s in the group with IAH, and was statistically significant (P = 0.003). In the group with IAH, the mean lactate was higher and the mean APP value was lower, with P values of 0.007 and 0.014, respectively. Mortality rate was 60% in the group with IAH and 31.2% in the group without IAH and there was no significant difference between the two groups (P = 0.156). The mean mesenteric rSO2 measurement was 44.9 ± 11.8% in the IAH group and 57.9 ± 8.8% in the without IAH group (P = 0.002). In the group with IAH, the mean ScvO2 was 59.6 ± 11.2% (P = 0.024) (Figure 2), the VIS was 40.4 ± 25.9 (P = 0.024) (Figure 3). The mean renal rSO2 value was 50.6 ± 12 (P = 0.004) in the IAH group. The comparison of mean mesenteric and renal rSO2 value in two groups is shown in Figure 4. In the group with IAH, 13 patients were receiving sedation–analgesia. The mean mesenteric rSO2 measurement was 42.8 ± 11.3% in patients with sedation–analgesia and 58.3 ± 1.59% in patients without sedation–analgesia (P = 0.076). 

**Table 2:** Admission diagnosis of study group.

Massive fluid resuscitation applied septic shock (n = 8)Intraabdominal sepsis (n = 2)Multiorgan dysfunction syndrome (n = 1)
ECMO (n = 4)
Multiple trauma (n = 3)
Pneumonia ventilated with high PIP pressures (n= 3)
Abdominal surgery (n = 10)
Pancreatitis
Cholestasis (n = 2)
Necrotizing enterocolitis (NEC)
Malrotation
Ileus
Hepatectomy
Wilm’s tumor Surrenal tumorHydatid cyst of the liver

**Table 3:** Demographics and clinic characteristics of patients.

Patient	With IAH group (mean ± SD)(median (min–max))	Without IAH group(mean ± SD)(median (min–max))	P-value
Age (month)	58.47 ± 53.148 (3–156)	52.75 ± 42.942 (2–132)	0.984
Body weight (kg)	13.7 ± 7.514.5 (3.7–25)	12.9 ± 8.113.5 (3.5–24)	0.599
PIM II	63.2 ± 13.568 (36–80)	63.8 ± 21.264 (28–98)	0.984
PRISM III	27. 8 ± 6.128 (17–39)	25.1 ± 8.324 (12–39)	0.281
PELOD	29.6 ± 10.332 (10–44)	23.2 ± 10.820 (12–44)	0.086
BUN (mg/dL)	29.4 ± 28.824 (0.53–119)	17.3 ± 13.912 (3–53)	0.175
Creatine (mg/dL)	1 ± 0.570.87 (0.4–2.6)	0.8 ± 0.750.51 (0.15–2.7)	0.072
Urine output (mL/kg/h)	5 ± 10.41.4 (5–42)	3.2 ± 2.13 (1–10)	0.140
Capillary refilling time (s)	3.8 ± 1.14 (2–6)	2.5 ± 0.72 (2–4)	0.003
MAP-mean (mmHg)	64.8 ± 16.260 (41.2–94.5)	75.4 ± 15.972.3 (56.7–110.7)	0.072
APP-mean (mmHg)	52.9 ± 16.546.7 (26.2–81)	66.9 ± 16.563.5 (47.5–104.2)	0.014
Lactate-mean (mmol/L)	7.5 ± 7.45.1 (1.7–31)	2.7 ± 1.32.1 (1.2–5.6)	0.007
mesenteric-rSO2-mean (%)	44.9 ± 11.844 (26.5–70.5)	57.9 ± 8.856.2 (45.7–73.5)	0.002
renal-rSO2-mean (%)IAP (mmHg)ScvO2 (%)VISEF (%)Hb (g/dL)	50.6 ± 1251.2 (25.7–67)11.8 ± 1.511.2 (10–15)59.6 ± 11.262 (38–76)40.4 ± 25.942 (0–83)51.4 ± 11.449 (30–72)9.2 ± 1.98.3 (7–13)	64.2 ± 9.764.7 (44.5–80.7)8.2 ± 1.28.8 (6.5–9.7)71.1 ± 12.272 (45–90)15.8 ± 24.30 (0–57)61.8 ± 10.865 (37–75)10.3 ± 1.610.5 (7–13)	0.004<0.0010.0240.0240.0120.093

**Figure 5:**
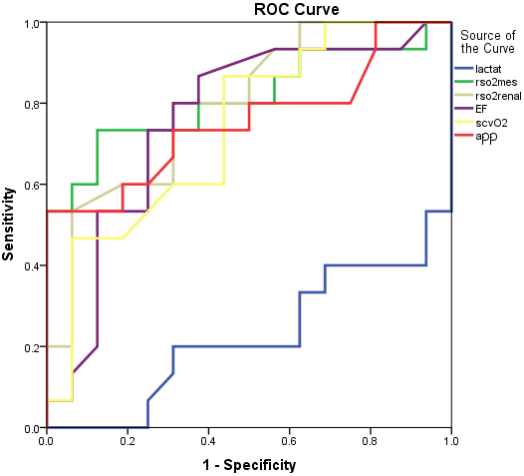
AUC of mean rSO2 mesenteric, rSO2 renal, APP, lactate, EF and ScvO2 levels in ROC analysis

In the correlation analysis there was a statistically significant, negative correlation between IAH and, renal rSO2 and mesenteric rSO2 parameters (respectively, r = 0.508, P = 0.004; r = 0.590, P < 0.001). We detected a statistically significant, positive correlation between IAH and mean lactate levels. ROC analysis was done with mean lactate, EF, ScvO2, renal rSO2, and mesenteric rSO2 parameters for IAH (Table 4). We observed that mesenteric rSO2 has the highest area under curve (AUC) to detect IAH with a 0.812 and a 0.652–0.973 95% confidence interval (P = 0.003) (73.3% sensitivity, 87.5% specificity, 84.6% positive predictive value (PPV), 77.8% negative predictive value (NPV), and 80.6% accuracy) (Figure 5). The cut-off value for mesenteric rSO2 was 50% and the cut-off value for renal rSO2 was 57.5% in our study.

**Table 4:** Results of ROC analysis.

ROC analysis	Cut-off value	Area under curve	%95 confidence interval	P-value	Sensitivity	Specifity	PPV	NPV	Accuracy
Mesenteric_rSO2_mean (%)	50.0	0.812	0.652–0.973	0.003	73.3	87.5	84.6	77.8	80.6
Lactate_mean (mmol/L)	2.8	0.777	0.609–0.945	0.009	80.0	62.5	66.7	76.9	70.9
Renal_ rSO2_mean (%)	57.5	0.798	0.642–0.954	0.005	80.0	68.8	70.6	78.6	74.1
EF (%)	58.5	0.760	0.583–0.938	0.013	73.3	75.0	75.0	73.3	74.1
ScvO2 (%)	71.0	0.738	0.561–0.914	0.024	86.7	56.2	65.0	81.8	70.9

Intraabdominal hypertension is associated with increased mortality and morbidity in pediatric intensive care units. Increased IAP causes a rise in the tension on the abdominal wall and causes impairment of perfusion and associated with poor outcomes in pediatric intensive care units [3]. Therefore, splanchnic blood flow may be reduced by the early response to global hypoperfusion and mesenteric rSO2 values rapidly decrease in IAH [14]. The recognition of IAH by pediatric intensive care physicians and taking the necessary precautions is very important to prevent progression to ACS and improve outcomes [15]. In our study we showed that the mesenteric rSO2 is a preferable and noninvasive tool for detecting IAH in critically ill pediatric patients. To the best of our knowledge, this is the first study which assesses the use of mesenteric and renal rSO2 measurements with NIRS in children to identify IAH. 

Conventional hemodynamic monitoring commonly used parameters are either age-dependent (heart rate, blood pressure), invasive (central venous pressure, central venous oxygen saturation) or laboratory parameters (serum lactate level, metabolic acidosis) [16]. Near-infrared spectroscopy is a noninvasive monitoring tool of local tissue oxygenation, it is a continuous method and provides real-time feedback to clinicians. Near-infrared spectroscopy has become a widely used noninvasive clinical monitor and appears to be a favorable, beneficial hemodynamic monitor which improves the care of critically ill pediatric patients and provides early warning for cerebral or somatic hypoperfusion [17]. Decreased perfusion leads to increased oxygen extraction which results in lower rSO2 measurements. In a retrospective study, Balakrishnan et al. [18] evaluated low somatic NIRS values at pediatric intensive care admission in addition to other hemodynamic monitoring tools for critically ill children (serum lactate, severity of illness scores, heart rate, systolic blood pressure, arterial oxygen saturation, lactate level, acidosis) and need for life saving interventions (cardiopulmonary resuscitation, ECMO, invasive or noninvasive ventilation, emergent surgery, emergent dialysis, need for fluid resuscitation >40 mL/kg, blood product transfusion or vasoactive medications). They found a positive correlation between low somatic NIRS oxygen saturation and need for life saving interventions and a higher mortality rate in low NIRS group compared to normal NIRS group. They suggested that somatic NIRS monitoring may identify children at high risk of medical instability. In our study, mesenteric rSO2, lactate, renal rSO2, EF, and ScvO2 were related to intraabdominal hypertension. However, the highest sensitivity, specificity, positive predictive value, negative predictive value and accuracy values were belong to mesenteric rSO2. Therefore, we believe that mesenteric rSO2 may be preferred to detect intraabdominal hypertension. Another preferred reason is that mesenteric rSO2 is noninvasive. In recent years there has been a noninvasive tendency in intensive care units. 

The patient population ranges from newborn to adolescent for pediatric intensivist. The thickness of abdominal wall varies with patient size and age. Abdominal wall thickness between the skin and the region of interest is important for the appropriate measurement of NIRS probes. Balaguru et al. [13] found better correlation between abdominal wall thicknesses and body weight than age. In the present study, we excluded patients over 25 kg to prevent inaccurate measurements of NIRS probes and we measured abdominal wall thickness by abdominal computed tomography in patients older than 6 years of age. In children, ultrasonography is used to measure abdominal wall thickness in various conditions [19–21]. We could also measure the abdominal wall thickness of our patients using ultrasonography. However, eight of our patients with diagnoses such as pancreatitis, trauma, ileus, liver hydatidosis, and intraabdominal mass had already undergone abdominal tomography imaging. Therefore, abdominal wall thickness measurements obtained from the abdominal tomography measurements were noted. 

In a PubMed-based literature review we could not find a study about the correlation between IAH and rSO2 in pediatric age group. Our study is the first to examine the relationship between IAH and perfusion markers. In a study including 19 neonates who underwent abdominal surgery, cerebral and renal rSO2 values were measured during surgery, and a decrease in cerebral rSO2-fixed renal rSO2 levels was found [22]. In the same study, at the postoperative period, an increase of 0.71% per hour was detected in renal rSO2 values. A significant correlation was found between renal rSO2 values and oxygen saturation, showing that a 1% increase in saturation caused a 1.5% increase in renal rSO2. There was no correlation between MAP and heart rate with renal rSO2. Our study results showed a statistically significant, negative correlation between IAH and, renal rSO2 and mesenteric rSO2 parameters, and a positive correlation between IAH and mean lactate levels.

Kaufman et al. [23] measured intramucosal gastric pH with gastric tonometer as well as abdominal rSO2 measurements in their study of patients who underwent congenital heart surgery and found a significant positive correlation between rSO2 and gastric pH. In the same study, they found a significant negative correlation between serum lactate levels and mesenteric rSO2 levels. They reported that splanchnic rSO2 has strong correlations with gastric pH, serum lactate, and ScvO2 and may serve as an index for systemic oxygenation and perfusion of patients in the intensive care unit after congenital heart surgery. In our study, we found significantly elevated lactate, significantly lower ScvO2, mesenteric rSO2 and APP levels in the IAH group.

Kim et al. [14] compared the abilities of cerebral, renal, and splanchnic rSO2 immediately after weaning from cardiopulmonary bypass to predict early postoperative outcomes. The study includes 73 children undergoing corrective or palliative cardiac surgery. Splanchnic rSO2 may be superior to cerebral and renal rSO2 in predicting an increased requirement for vasoactive inotrops, prolonged mechanical ventilation, and a longer postoperative hospital stay for children [14]. No mortality occurred in their study. Our study showed a significantly higher VIS level in the IAH group. The mortality rate was 60% in our with IAH group but this situation was not statistically significant.

Stapleton et al. [24] performed mesenteric rSO2 measurements in a neonatal case with NEC, and found a significant decrease in rSO2 values. A significant increase in mesenteric rSO2 values was detected with intravenous antibiotic treatment and discontinuation of the feeding. However, IAP was not measured in this study. Zabaneh et al. [25] monitored the mesenteric rSO2 measurements in the twelve-day-old twins, one of whom had NEC and the other one was healthy. They found significantly lower mesenteric rSO2 measurements in the NEC twin, compared to the healthy one. Measurements of rSO2 of the NEC twin were elevated to the same level as the healthy twin after surgery. Many studies such as this note the importance of abdominal NIRS monitoring in tissue oxygenation in neonates with NEC [26,27]. 

The clinical and laboratory findings of poor perfusion might be affected by other clinical conditions associated with the patient such as renal failure, mechanical ventilation, sedation–analgesia, need for vasoactives or vasopressor agents [28]. When we evaluate for the sedation–analgesia in the group with IAH, there was no significant difference in the mean mesenteric rSO2 measurement between patients with sedation–analgesia and without sedation–analgesia.

In conclusion, IAH and ACS are common problems with high morbidity and mortality in critically ill children. Judging by our study results and the other studies in the literature, we can say that IAH is a condition of poor perfusion and decreasing in mesenteric rSO2 levels is important for predicting IAH and poor clinical outcomes in critically ill pediatric patients. We think that, compared to other perfusion markers, rSO2 can be easily used in the detection of IAH in terms of noninvasive and continuous measurement. To the best of our knowledge there is no similar study in the pediatric literature and the study is important in this direction. 

This study had some limitations. It was a study from a single medical center with a small sample and although mesenteric and renal rSO2 values were continuously monitored, statistical analysis was performed with recorded values at 6-h intervals. Further studies conducted using continuous measurements are needed to determine the association between target mesenteric rSO2 values and clinical outcomes in critically ill children. It may be useful to study with larger patient groups in this regard. 

## Acknowledgments

We would like to thank the patients’ parents for their permission for using their data in this clinical trial.

## Contribution of authors

Dr. Nagehan Aslan designed the data collection instruments, enrolled the patients, collected the data, performed echocardiography, drafted the initial manuscript and approved the final manuscript as submitted.

Dr. Özden Özgür Horoz conceptualized and designed the study, coordinated and supervised data collection and approved the final manuscript as submitted

Dr. Dinçer Yıldızdaş and Dr. Awni AL-SUBU critically reviewed the manuscript and approved the final manuscript as submitted.

Dr. Yasemin Çoban coordinated and supervised data collection.

Dr. Yaşar Sertdemir performed statistical analyses.

## References

[ref0] (u2ddb). Pediatric guidelines sub-Committee for the World Society of the abdominal compartment syndrome. Intra-abdominal hypertension and the abdominal compartment syndrome: updated consensus definitions and clinical practice guidelines from the world Society of the Abdominal Compartment Syndrome. Intensive Care Medicine.

[ref1] (2008). What is the normal intra-abdominal pressure in critically ill children and how should we measure it?. Critical Care Medicine.

[ref2] (2016). An Update on Intra-abdominal Hypertension and Abdominal Compartment Syndrome in children. Journal of Pediatric Critical Care.

[ref3] (u2dd9). The prevalence of and factors associated with intra-abdominal hypertension on admission day in critically ill pediatric patients: a multicenter study. Journal of Critical Care.

[ref4] (2011). Abdominal compartment syndrome: focus on the children.

[ref5] (2016). Incidence and prognosis of intraabdominal hypertension and abdominal compartment syndrome in children. Journal of Pediatric Surgery.

[ref6] (u2dd8). Intra-abdominal hypertension; prevalence, incidence and outcomes in a low resource setting; a prospective observational study. World Journal of Emergency Surgery.

[ref7] (u2dd6). Abdominal perfusion pressure: a superior parameter in the assessment of intra-abdominal hypertension. Journal of Trauma.

[ref8] (2006). Cardiovascular implications of elevated intra-abdominal pressure.

[ref9] (2011). Tissue oxymetry or near infrared spectroscopy (NIRS). IRBM News.

[ref10] (2010). Near-infrared spectroscopy (NIRS) monitoring in contemporary anesthesia and critical care. Acta Anaesthesiologica Belgica.

[ref11] (2010). Vasoactive-inotropic score as a predictor of morbidity and mortality in infants after cardiopulmonary bypass. Pediatric Critical Care Medicine.

[ref12] (u2dd5). Computed tomography scan measurement of abdominal Wall thickness for application of near infrared spectroscopy probes to monitor regional oxygen saturation index of gastrointestinal and renal circulations in children.

[ref13] (u2dde). Splanchnic oxygen saturation immediately after weaning from cardiopulmonary bypass can predict early postoperative outcomes in children undergoing congenital heart surgery. Pediatric Cardiology.

[ref14] (u2dd3). Incidence, risk factors, and prognosis of intra-abdominal hypertension in critically ill children: a prospective epidemiological study. Journal of Intensive Care Medicine.

[ref15] Multiple organ dysfunction syndrome in children. Pediatric Critical Care Medicine 2003; 4.

[ref16] (2008). Use of near-infrared spectroscopy as a physiologic monitor for intra-abdominal hypertension. Journal of Trauma.

[ref17] (2018). Low near infrared spectroscopic somatic oxygen saturation atadmission is associated with need for lifesaving interventions among unplanned admissions to the pediatric intensive care unit. Journal of Clinical Monitoring and Computing.

[ref18] (2019). Model to estimate abdominal wall thickness in children undergoing placement or replacement of gastrostomy devices. Journal of Pediatric Surgery.

[ref19] (2015). Lateral abdominal muscle size at rest and during abdominal drawing-in manoeuvre in healthy adolescents. Manuel Therapy.

[ref20] (2019). Frequency of technical success of two-dimensional ultrasound shear wave elastography in a large pediatric and young adult cohort: a clinical effectiveness study. Pediatric Radiology.

[ref21] (u2dcf). Monitoring Cerebral and Renal Oxygenation Status during Neonatal Digestive Surgeries Using Near Infrared Spectroscopy. Frontiers in Pediatrics.

[ref22] (u2dcd). Correlation of abdominal site near-infrared spectroscopy with gastric tonometry in infants following surgery for congenital heart disease. Pediatric Critical Care Medicine.

[ref23] (2007). Mesenteric oxygen desaturation in an infant with congenital heart diasease and necrotizing enterocolitis. Texas Heart Institute Journal.

[ref24] (2011). Mesentric oxygen saturations in premature twins with and without necrotizing enterocolitis. Pediatric Critical Care Medicine.

[ref25] (u2dce). Splanchnic near-infrared spectroscopy and risk of necrotizing enterocolitis after neonatal heart surgery. Pediatric Cardiology.

[ref26] (u2dcc). Near-Infrared Spectroscopy to predict the Course of Necrotizing Enterocolitis. PLoS One.

